# The speed of race

**DOI:** 10.1093/scan/nsad076

**Published:** 2023-12-15

**Authors:** Peter de Lissa, Pauline Schaller, Roberto Caldara

**Affiliations:** Eye and Brain Mapping Laboratory (iBMLab), Department of Psychology, University of Fribourg, Fribourg 1700, Switzerland; Eye and Brain Mapping Laboratory (iBMLab), Department of Psychology, University of Fribourg, Fribourg 1700, Switzerland; Eye and Brain Mapping Laboratory (iBMLab), Department of Psychology, University of Fribourg, Fribourg 1700, Switzerland

**Keywords:** face processing, race, other-race face categorization advantage, N170, P1

## Abstract

When asked to categorize faces according to ‘race’, people typically categorize other-race faces faster than faces belonging to their own race. This ‘Other Race Categorization Advantage’ is thought to reflect enhanced sensitivity to early visual signals characteristic of other-race faces, and can manifest within 200 ms of face presentation. However, recent research has highlighted the importance of signal intensity in this effect, where visual-degradation of the face images significantly enhances the effect and exposes a behavioural threshold at very low levels of visual quality where other-race visual signals are able to be perceived while same-race signals are not. The current study investigated the effect of signal intensity in race categorization processes in the brain through electroencephalography and in accuracy/reaction times. While replicating the previously observed enhancement of the other-race categorization advantage, we also found enhanced sensitivity to other-race faces in early P1 peaks, as well as later N170 and N250 peaks. These effects, however, related to the varying levels of signal intensity in the face stimuli, suggesting that race categorization may involve different types of perceptual and neural processes rather than one discrete process. The speed at which race is perceived depends on the intensity of the face signal.

## Introduction

The social intra- and interpersonal consequences of the perception of human race are often deep and wide-reaching. However, the early low-level perceptual dimensions that lead to perception of race and the plethora of social consequences are, however, not yet as clarified as it should be, given the range of the effects that can stem from this fast visual categorization. The salience of race in the early perceptual processes has become clear, with behavioural tasks revealing a clear bias towards the recognition of ‘other-race’ visual signals in faces, leading to faster categorization of other-race faces when discriminating race (Valentine and Endo, 1992; Levin, 1996, 2000; Caldara *et al.*, 2004; Zhao and Bentin, 2008, 2011; Ge *et al.*, 2009; Feng *et al.*, 2011). Termed the Other Race Categorization Advantage (ORCA), such an enhanced sensitivity has the potential to bias visual attention when people are confronted with competing face stimuli where faster perception of race in other-race faces may lead to enhanced attention and focus in various contexts involving saccadic selection of face targets (de Lissa *et al.*, 2021). However, a recent study has shown that not only can enhanced sensitivity to other-race faces lead to reaction time (RT) differences when categorizing race, but also differences in the fundamental ability to perceive and categorize race at all. When images of same- and other-race faces are systematically degraded in visual quality, there is a distinct threshold difference where same-race faces are no longer reliably able to be categorized in terms of race, while other-race faces are (de Lissa *et al.*, 2022). Such an effect was interpreted to relate to a differential sensitivity to the visual patterns characteristic of different race faces, where an overall speed difference is apparent in typical, full-structure face images. However, when the intensity of the visual signals of the faces is systematically decreased, it leads to a point where the visual signals characterizing same-race faces are not reliably perceived while those for other-race faces are. Such an effect has the potential for profound social effects when visual quality is markedly reduced, such as in low-resolution photo/video or in live situations where low ambient light makes visual perception more difficult. However, the finding also provides an opportunity to probe further the neural activations relating to race categorization.

Event-related-potential (ERP) studies investigating race modulation of neural responses have found mixed effects in the early visual P1 and N170 peaks. While the P1 peak is generally associated with low-level stimulus attributes (Regan, 1989), a number of studies have found race modulation of this early brain potential, although with mixed results. While some have found larger activations for own-race faces (Caharel *et al.*, 2011; Herzmann, 2016; Giménez-Fernández *et al.*, 2020), others have reported the opposite effect of larger P1 peaks to other-race faces (Hahn *et al.*, 2012; Wang *et al.*, 2020), with others reporting no P1 modulation of race at all (Senholzi and Ito, 2013; Vizioli *et al.*, 2010a,b, see Serafini and Pesciarelli, 2023 for review.) In a mixed result, He *et al.* (2009) found a modulation of P1 by race, with Black faces eliciting larger peaks than White faces (own race in the participant sample), which were in turn higher than Asian faces. The authors noted that the effect may have arisen from low-level visual property differences between the face stimuli such as skin tone (which were not balanced), but also speculated that there may exist an interaction between different facial features of various races and the deployment of visual attention. A clarification of the contribution of race processing to the P1 would move towards determining the timing of race perception in stimuli that are balanced for low-level visual properties, particularly in light of the behavioural results indicating that race categorization can occur in as little as 200 ms after face presentation (de Lissa *et al.*, 2021).

Similar to the conflicting pattern of P1 results, studies investigating race-modulation in early N170 responses have found mixed results, with a number of studies finding no effect for upright faces (Caldara *et al.*, 2003, 2004; Lv *et al.*, 2015; Proverbio and De Gabriele, 2019; Sun *et al.*, 2014; Tanaka and Pierce, 2009; Vizioli *et al.*, 2010a,b; Wiese *et al.*, 2009), others finding an enhancement of N170 amplitude to other-race faces (Walker *et al.*, 2008; Stahl *et al.*, 2010; Caharel *et al.*, 2011; Ofan *et al.*, 2011; Wiese *et al.*, 2014; Anzures and Mildort, 2021) and another finding the opposite pattern (Ito and Urland, 2005; Vizioli *et al.*, 2010a). Senholzi and Ito (2013) provided clear evidence that the N170 effect is related to the task instructions, where race categorization led to enhanced N170 peaks for same-race faces while face recognition led to enhanced N170s to other-race faces. However, Wiese (2013) found the opposite pattern where other-race faces elicited larger N170 peaks in a race categorization task. Accordingly, it is difficult to confidently state whether the N170 is specifically modulated by race, or if it is then how. Part of the issue might relate to how race processing itself contributes to the N170 peak, relative to the other structural-encoding processes taking place at this time (Bentin *et al.*, 1996; Rossion *et al.*, 1999; Eimer, 2000). A relatively small, nuanced activation difference due to race perception might be overwhelmed in size by other co-occurring processes contributing to N170 activity, and consequently may not manifest as clearly or as reliably by contrast.

More robust effects have been observed in the later N250 peaks, with larger activations found for other race faces (Sun *et al.*, 2014; Wiese *et al.*, 2014; Herzmann, 2016), interpreted as reflecting greater resources required for individuating these faces relative to same-race faces due to differences in experience (Herzmann, 2016) and differences in face-related semantic activation (Caldara *et al.*, 2004). However, in light of evidence from saccadic-response paradigms suggesting that race categorization behavioural responses can reliably occur prior to the N250 time-range (de Lissa *et al.*, 2021), it is possible that earlier perceptual processes (P1 and N170) give rise to the later ones. The lack of distinct race modulation patterns in these early neural responses therefore might suggest a poor signal-to-noise ratio for such signals within the backdrop of other neural processes in ERP recordings.

However, the race-in-noise paradigm may provide a useful approach to address this potential issue. By presenting faces which have been systematically phase-scrambled to decrease the amount of visual information contained in face images, we can see not only the enhanced behavioural sensitivity to other race faces but potentially also any neural differences associated with this race perception. Specifically, a critical threshold was observed in de Lissa *et al.* (2022) where participants were unable to reliably categorize race when presented with significantly degraded same-race faces, and yet they retained the ability to perceive race-signals in other race faces. At this critical point, we might find clear neural expressions of the sensitivity to other race faces that is often found in purely behavioural paradigms. The principal of visually degrading stimuli has previously been used to observe how face perception and sensitivity to face stimuli manifests neurally as face structure is lost within phase-scrambled backgrounds. By parametrically reducing the visual information contained in face images in a face discrimination task, Rousselet *et al.* (2008) found that the neural responses between 100 and 200 ms (encompassing the P1 and N170 responses to faces) decreased systematically until the structure was completely degraded leaving no face-sensitive neural response at all. Accordingly, we should expect a similar reduction in N170 response to face structure in a race-in-noise paradigm while preserving race categorization ability. By reducing the other encoding processes leading to N170 activity, we should hope to find a clearer contribution of the visual race signal processes. Further, the results of de Lissa *et al.* (2022) suggest that the ORCA itself is enhanced as face structure is degraded, leading to a maximally enhanced sensitivity to other-race faces at 25–30% structure level (phase-coherence level). We may thus be able to view the neural activity relating to race categorization processes more clearly when the behavioural difference is at its peak. Finally, and perhaps most critically, the onset of other-race sensitivity in both Swiss and Japanese observers was observed at 20% structure level (de Lissa *et al.*, 2022), where participants were able to reliably categorize the other-race faces by race whereas the same-race faces were still categorized at chance level. Neural responses at this behavioural threshold may provide insight into the presence *vs* absence of race perception—when visual signal is reduced to a critical threshold, the visual signals of other-race faces remain salient enough for race categorization to be reliably performed while same-race face signals are no longer perceived. Therefore, in the current study, we sought to implement the race-in-noise paradigm to determine whether the behavioural threshold at which race processing differences first manifest also leads to early N170 differences indexing the heightened sensitivity to other-race face signals.

### Methods

The Human Ethics Committee at the University of Fribourg approved the methods and procedure used in this study. All participants provided written informed consent in accordance with the Declaration of Helsinki. All data used in statistical analyses and represented in figures are available online on the Open Science Framework repository at https://osf.io/sx6b2.

### Participants

A total of 38 participants (West European) were recruited from the student pool at the University of Fribourg. While de Lissa *et al.* (2022) used a cross-cultural design with both Swiss and Japanese participants, the current study utilized only a sample of Swiss participants to focus directly on the clear enhancement of the ORCA in this cultural group.

One participant dataset was rejected due to severe electroencephalography (EEG) artefacts due to movement, and one dataset was rejected due to improper engagement with the behavioural task (chance performance through all coherence levels). A total of 36 participant datasets were thus statistically analysed in the study (M_age_ = 23.7 years, SD_age_ = 4.5, 35 right-handed, 33 female). All participants reported having normal or corrected-to-normal vision, and were given research participation credit for their respective studies. The sample size in the current study was informed by the effect sizes from EEG studies investigating other-race categorization and identity effects in terms of neural responses (Senholzi and Ito, 2013; Wiese, 2013), with calculations based on the smallest effect size (0.19) at alpha = 0.05 and power = 0.8. These calculations suggested a sample size of *N* = >35 to be a conservative approach.

### Stimuli and procedure

**Fig. 1.**
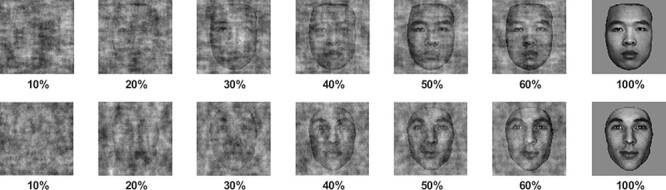
Examples of EA and WE face stimuli used in the study, ranging from 10% structural coherence through to 60%, and at 100% full coherence. The WE and EA were equated for low-level visual qualities.

The stimuli consisted of 20 grayscale face images of 10 European and 10 Asian identities (equal numbers of male and female). The images were neutral expression portrait photographs of Belgian (West European—WE) and Chinese (East Asian—EA) students aged from 18 to 25 years, utilized in a previous study investigating the impact of the systematic removal of visual information on race categorization judgements (de Lissa *et al.*, 2022), as well as other studies investigating race perception (Michel *et al.*, 2006a, 2006b; de Lissa *et al.*, 2021). The faces were cropped to exclude ears and hair, and were matched for amplitude spectra, luminance and contrast using the SHINE toolbox (Willenbockel *et al.*, 2010). Systematic degradation of the structure of the face images was achieved through a phase-scrambling technique which randomizes the location of all spatial frequencies in the images while preserving amplitude spectra across orientations and spatial frequencies (Honey *et al.*, 2008). In order to adapt the array of stimuli from the preceding behavioural study (de Lissa *et al.*, 2022) to an ERP paradigm utilizing more trials per condition, a subset of stimuli were used to test critical levels of coherence from 10% (where participants had been unable to reliably categorize race) through to 60% coherence (where participants exhibited an accuracy ceiling), in 10% coherence steps. An additional 100% stimulus coherence condition was added to provide a baseline from which to view face and race processing when stimulus quality was not degraded (see Figure 1).

Participants were presented with WE and EA faces possessing systematically varied amounts of phase coherence, and were instructed to categorize the race of the faces through a keyboard. The instructions noted that participants might find some conditions impossible to categorize, in which case they could make a random guess to avoid pressing the same response button. The experimental stimuli (11.6˚ × 11.6˚ in viewing angle) were presented on a 24-inch VIEWPixx/3D monitor (1920 × 1080 pixels, 120 Hz refresh rate) at a distance of 70 cm, and presented through Experiment Builder (v1.10.1630) software. Trials commenced with a blank screen presented from between 2100 and 2500 ms. A central fixation cross was then presented for 500 ms, followed by a 200 ms blank screen before the target face stimuli were presented for 200 ms. The target faces were replaced with a black screen until the participant pressed a key. There were 840 trials in total, with 60 trials for each of the 2 stimulus race types (EA and WE) and 7 coherence conditions (10%, 20%, 30%, 40%, 50%, 60% and 100%). Breaks were implemented every 60 trials.

### EEG recording

EEG was recorded with a Biosemi Active-Two amplifier system using 128 Ag/AgCl electrodes sampling at 1024 Hz. EEG data were processed offline though EEGLAB (v2021.1) running on MATLAB 2021a. Bandpass filtering (0.1–30 Hz) was performed before epochs were formed (−100 to 1000 ms), followed by baseline-correction to the average voltage from −100 to 0 ms. Eye-blink artefact correction was achieved through Independent Component Analysis (ICA, Delorme and Makeig, 2004). Bad EEG channels were excluded from ICA analysis, and interpolated subsequently. The EEG epochs were then re-referenced using a common-average reference, and epochs were rejected if voltages exceeded ±100 μV within the first 500 ms after stimulus presentation. ERP averages were formed for each condition and for each participant.

### Data processing & analysis

#### Behavioural data

Participant datasets were assessed for inclusion/exclusion before statistical analysis. Trials with behavioural response times shorter than 100 ms and longer than 2000 ms were excluded from the datasets. Participants with lower than 75% overall accuracy in the full-structure 100% coherence conditions were rejected. This process rejected participants who were not engaged in the task and made single-key responses. These inclusion criteria excluded 2 datasets from the 38 participants, leaving 36 behavioural and EEG datasets for analyses. The behavioural study on which the current study was based utilized an additional rejection criteria where participants exhibiting higher than 75% accuracy in a 0% coherence condition were excluded from analysis (de Lissa *et al.*, 2022). We did not utilize this additional criteria in the lowest coherence level (10%) in the current study as the preceding study had already established that the 20% coherence condition was the behavioural threshold for participants’ ability to categorize EA faces, and the bias to make an EA or WE response when guessing is conceptually unrelated to this threshold.

Condition means were calculated for each participant for accuracy (correct responses divided by accepted trials) and RT (of correct trials). Accuracy and RT data were separately subjected to Bayes Factor analyses for an effect of stimulus race at each of the seven coherence levels. Bayes Factor thresholds representing sufficient evidence to denote the presence of an effect in the accuracy and RT data (difference in means) were taken as *BF*_10_ = >3, and at *BF*_10_ ≤ 0.33 for evidence supporting the null hypothesis. Evidence for the null hypothesis is presented in the figures containing Bayes Factor analyses but is not discussed for brevity.

#### EEG data

**Fig. 2.**
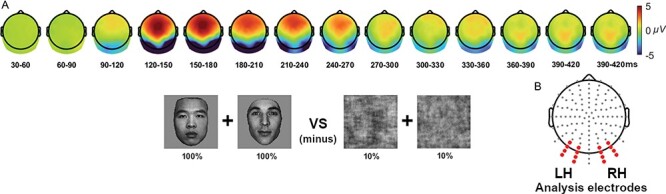
A topographical comparison of the effect of face sensitivity (all 100% faces minus all 10% faces) confirmed bilateral occipito-temporal scalp locations as the areas of maximal difference from an early N170 period through to an early N250 period. Accordingly, two bilateral electrode clusters (B) were chosen for Bayes Factor analyses in the seven coherence levels (10–60%, and 100%).

After the EEG pre-processing steps, the participant ERP averages for the 100% and 10% coherence levels were compared to confirm the spatial location of face-sensitivity in our electrode montage and using a common-average reference, which corresponded to left and right occipito-temporal scalp regions (see Figure 2A) over the early N170 and later N250 time ranges corresponding to scalp regions common to N170 activations found in previous studies (Joyce and Rossion, 2005). Two occipito-temporal electrode clusters of eight sensors each were selected to represent the left and right hemisphere face-sensitive scalp areas encircling the maximal N170 peak electrodes (Rousselet *et al.*, 2004; Kuefner *et al.*, 2010) for statistical analyses (see Figure 2B, noted in red). Bayes Factor analyses were performed on the average voltages in these left and right hemisphere clusters, and topographic maps of both voltage differences and *BF*_10_ evidence levels were computed to provide further insight into the specific location of effects found in the cluster analyses.

Comparisons were performed for each time sample (0–420 ms) in the left and right hemisphere clusters. The time range of the analyses was selected to avoid motor-preparation activity contaminating the EEG and confounding the interpretation of ERP data. To lessen the likelihood of false-positives, the evidence boundary for reporting sufficient evidence to denote an effect of a difference between conditions was set at *BF*_10_ = >10, corresponding to strong evidence (Schönbrodt and Wagenmakers, 2018; Stefan *et al.*, 2019). Evidence in favour of the null hypothesis (comparable means) remained at *BF*_10_ ≤ 0.33. Statistical analyses were conducted in RStudio (2021.09.2) using the BayesFactor package (0.9.12–4.3, Morey *et al.*, 2018). Finally, scalp maps of Bayes Factor values are presented to depict the intensity of evidence at each electrode to complement the left and right mean electrode cluster analyses (Huber-Huber *et al.*, 2019).

## Results

### ORCA—RT and accuracy

The analyses of race categorization RTs showed that at 10% coherence there was no conclusive evidence of difference or similarity between mean EA (843 ms) and WE (823 ms) faces (*BF*_10_ = 0.429). At 20% coherence level, an extreme evidence for an ORCA effect was observed (*BF*_10_ = 1.14 × 10^3^), with an average RT of 784 ms for EA faces while average WE face RT remained high at 838 ms. The RT ORCA peaked at 30% coherence level (*BF*_10_ = 1.58 × 10^6^) and continued in the 40% (*BF*_10_ = 2.15 × 10^3^), 50% (*BF*_10_ = 7.15 × 10^4^) and 60% (*BF*_10_ = 435.9) coherence levels. At 100% coherence, not only was evidence of a difference not observed, but there was moderate evidence that the EA and WE face categorization RTs were the same (*BF*_10_ = 0.204).

The analyses of race categorization accuracy revealed evidence that WE faces were more accurately categorized than EA faces at 10% (*BF*_10_ = 291.8). At 20%, this difference reversed, with EA faces (82.6%) being more accurately categorized than WE faces (70.4%; *BF*_10_ = 625.2). At 30% coherence, evidence of an advantage for EA faces peaked (96.8% *vs* 84.7%; *BF*_10_ = 5.84 × 10^3^), which was also observed but to a much smaller degree at 40% coherence (96.8% *vs* 91.4%; *BF*_10_ = 13.6). At 50% coherence, there was no clear evidence of difference or similarity in accuracy between the EA (97.1%) and WE (94.6%) faces. At 60% coherence, there was substantial evidence that the EA and WE faces were categorized at the same accuracy (96.5% *vs* 95.8%; *BF*_10_ = 0.179). At 100%, there was again no evidence for similarity or difference between the EA and WE faces (96.5% *vs* 97.8%; *BF*_10_ = 0.372).

**Fig. 3.**
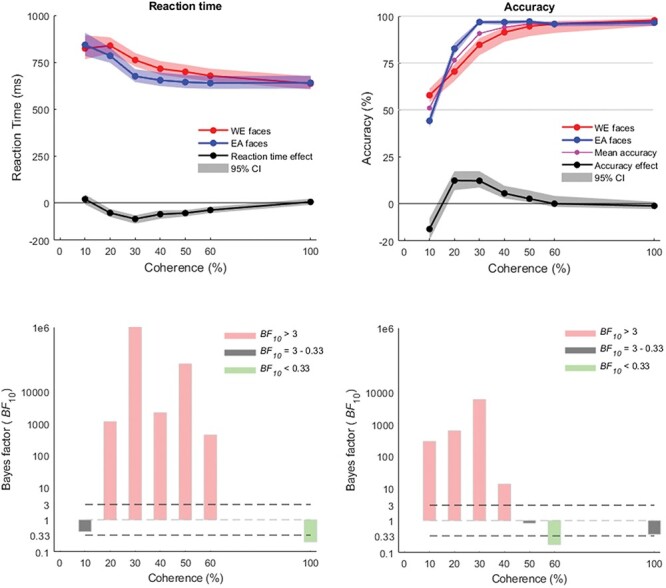
The reaction time profiles indicate an enhanced other-race categorization advantage arising at 20% coherence and continuing through to 60%. Accuracy similarly showed an other-race advantage at low coherence levels, excepting for the 10% condition. Shaded areas represent bootstrapped 95% confidence intervals.

The evidence of an advantage for WE faces at 10% coherence (57.8%) appears to come at the cost of accuracy for EA faces (44.2%), with overall accuracy at this level of coherence being 50.1% (see Figure 3). A d’ analysis of accuracy at 10% of the WE condition (which incorporates incorrect EA responses as WE false alarms) suggested very low discriminability (mean d’ = 0.034) and a bias towards WE responses (mean C = 0.18). Coupled with the lack of RT effect in the 10% coherence condition, it is likely that this accuracy effect was due to a bias of the participants to respond ‘WE’ when guessing.

### ORCA—EEG

#### 10% coherence

**Fig. 4.**
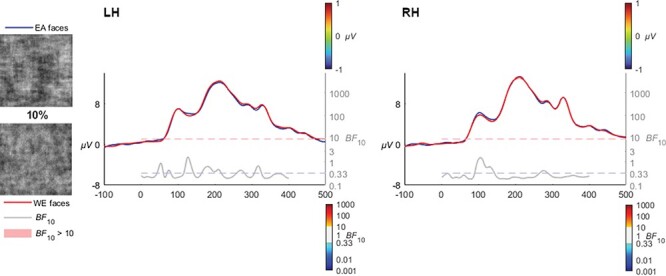
At 10% coherence levels, the EA and WE faces did not elicit any difference in neural response in the left or right electrode clusters. No scalp voltage topographies of voltage differences are illustrated as no Bayes Factor evidence (BF_10_ > 10) was found at this level of visual degradation.

At 10% coherence, no evidence of voltage differences was apparent, with left and right activation being mostly comparable (Figure 4).

#### 20% coherence

**Fig. 5.**
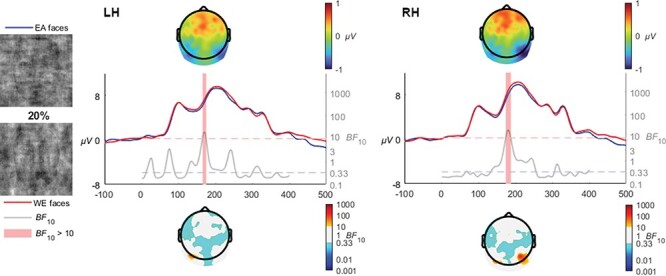
At the 20% coherence level, EA faces produced more negative voltages in the late N170 time ranges in both the left and right electrode clusters. Scalp voltage topographies (top) reflect EA minus WE voltages, while the scalp Bayes Factor topographies (bottom) reflect the regions where evidence of a difference (BF_10_ > 10, also reflected in the shaded periods in the waveforms) or of comparability (BF_10_ < 0.33) were observed.

At the 20% coherence level (the level at which both race categorization accuracy and RT effects emerged for the EA faces), an enhanced activation for EA faces was observed in the late N170 time range (see Figure 5) in both the left [*BF*_10_ > 10 from 161 to 170 ms, mean *BF*_10_ = 16, max *BF*_10_ = 18 at 166 ms] and the right [*BF*_10_ > 10 from 170 to 183 ms, mean *BF*_10_ = 17, max *BF*_10_ = 22.5 at 177 ms] hemispheres.

#### 30% coherence

**Fig. 6.**
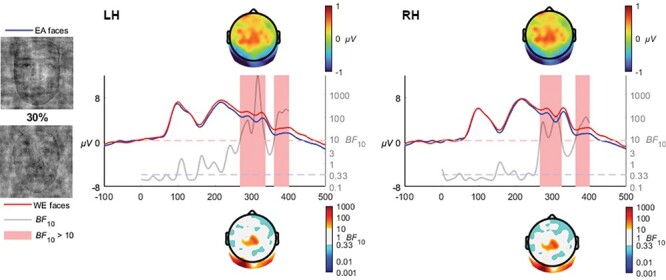
At 30% coherence, the enhanced amplitude to EA faces shifted later in time, corresponding with an N250 time range, manifesting in both the left and right hemisphere.

At 30% coherence, the N250 enhancement for EA faces continued to emerge strongly in both left [*BF*_10_ > 10 from 262 to 328 ms, mean *BF*_10_ = 757.5, max *BF*_10_ = 6911.9 at 309 ms] and right [*BF*_10_ > 10 from 260 to 316 ms, mean *BF*_10_ = 47.7, max *BF*_10_ = 114.4 at 303 ms] hemispheres. Only the strongest evidence in the left and right hemispheres is reported; however, there were additional periods of difference in the N250 range (see Figure 6).

#### 40% coherence

**Fig. 7.**
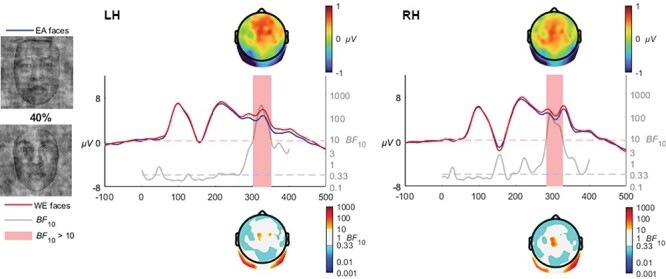
The enhanced N250 voltages continued in the 40% coherence level in both left and right hemispheres.

At 40% coherence, the enhanced N250 to EA faces were observed in both left [*BF*_10_ > 10 from 295 to 343 ms, mean *BF*_10_ = 128.9, max *BF*_10_ = 357.8 at 317 ms] and right [*BF*_10_ > 10 from 276 to 319 ms, mean *BF*_10_ = 64, max *BF*_10_ = 158 at 287 ms] hemispheres (see Figure 7).

#### 50% coherence

**Fig. 8.**
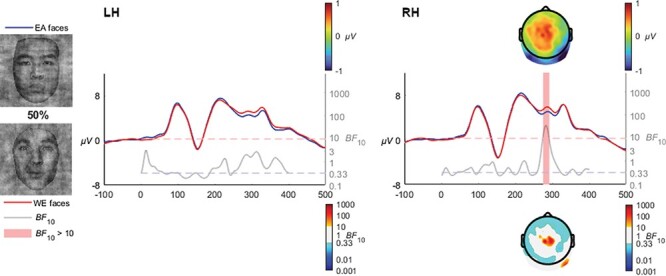
While an N250 EA effect was still apparent in the right hemisphere, it was shorter and not observed in the left hemisphere.

At 50% coherence, the earlier differences were no longer apparent (see Figure 8); rather, the later N250 enhancement for EA faces endured in the right hemisphere (RH) [*BF*_10_ > 10 from 269 to 284 ms, mean *BF*_10_ = 24.9, max *BF*_10_ = 36.7 at 276 ms].

#### 60% coherence

**Fig. 9.**
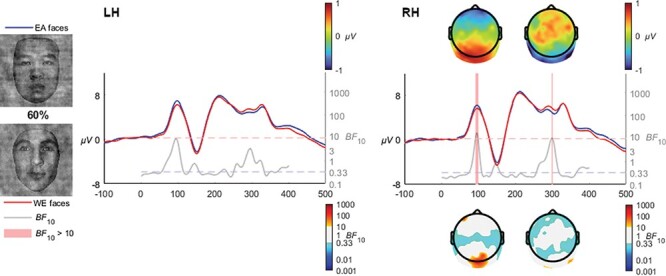
At 60% coherence, the later N250 effect (EA > WE) barely reached the BF_10_ evidence threshold in the right hemisphere, while an earlier effect was also observed where EA faces elicited larger P1 amplitudes than the WE faces.

The comparison at 60% coherence showed a brief enhancement of the early P1 to EA faces in the right hemisphere [*BF*_10_ > 10 from 90 to 97 ms, mean *BF*_10_ = 14.5, max *BF*_10_ = 17.8 at 94 ms]. EA faces also elicited greater RH N250 activation [*BF*_10_ > 10 from 291 to 293 ms, mean *BF*_10_ = 10.6, max *BF*_10_ = 10.8 at 292 ms] in the right hemisphere, albeit for only a very short duration (see Figure 9).

#### 100% coherence

**Fig. 10.**
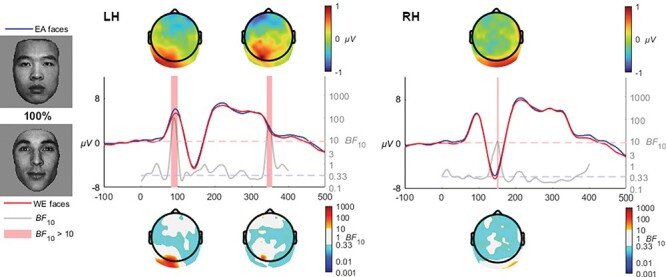
EA faces elicited a larger P1 in the left hemisphere, whereas WE elicited larger N170 and N250 time range activations in the right and left hemispheres, respectively.

The comparison of the EA and WE faces at 100% (full structure) coherence revealed an early enhanced P1 peak in the left hemisphere (LH) cluster for the EA faces [*BF*_10_ > 10 from 80 to 97, mean *BF*_10_ = 58.3, max *BF*_10_ = 106.6 at 88 ms] (see Figure 10). The N170 voltage in the RH however was larger for WE faces compared to EA [*BF*_10_ > 10 from 146 to 149 ms, mean *BF*_10_ = 10.8, max *BF*_10_ = 11.4 at 147 ms]. Similarly, the WE faces elicited greater LH voltage in the N250 time range [*BF*_10_ > 10 from 333 to 347 ms, mean *BF*_10_ = 32.6, max *BF*_10_ = 50.3 at 340 ms].

## Discussion

The overall pattern of the ERP results suggests that there are different stages or types of sensitivity to visual race signals depending on the quality of the visual stimulus, ranging from very early P1 and N170 effects through to later N250 effects which are more commonly found for recognition/individuation processes. Behaviourally, we observed that systematic reduction in image phase coherence enhanced the categorization advantage for other-race faces relative to same race faces in both RT and accuracy, peaking at 30% phase coherence. This pattern replicates the behavioural results of de Lissa *et al.* (2022), although the current study utilized a subset of coherence steps to make the testing time in the ERP paradigm more feasible. An accuracy effect was, however, observed in the 10% phase coherence condition in the current study, contrasting with the earlier findings where accuracy for both same- and other-race faces remained essentially at chance levels from 0% through to 15% coherence levels. However, as noted in section ‘ORCA—RT and accuracy’, the combination of the average response accuracy being approximately 50% chance level, the *d’* analysis result and the absence of a RT effect at the 10% coherence level suggests that there was a bias to respond ‘WE’ when guessing. As the response keys were counter-balanced, this is unlikely to be due to hand-dominance effects. It is reasonable to conjecture that such a pattern might emerge when participants do exhibit greater accuracy in one condition compared to another, so that when there is ambiguity, they may be more likely to choose the race they are less confident about, which in this case would be the WE condition. However, clarification of this particular effect might best be addressed by amending the paradigm to include a third ‘unsure’ response key in future studies.

Turning to the ERP analyses, the 10% coherence condition did not show any observable evidence of a stimulus race processing difference, which corresponds to the lack of RT effects and the average accuracy at chance level. The emergence of bilateral occipito-temporal effects at the 20% coherence level corresponded with clear accuracy and RT effects, reflecting enhanced sensitivity to other-race visual signals. The timing of these differences in the waveforms suggests they involve the later N170 activations rather than the peaks themselves. While approaching the P200 in time, which has been found to index distance-to-norm in terms of face typicality (Schweinberger and Neumann, 2016; Wuttke and Schweinberger, 2019) and sensitivity to other-race faces in face recognition (Anzures and Mildort, 2021) and repetition suppression (Wang and Han, 2021) paradigms, it is perhaps more likely that the extent of the phase-scrambling to a 20% coherence level created a very difficult condition for the visual system to interpret (see Figure 5 for an example of the stimuli at this coherence level), which may introduce variability in the latency of the N170 akin to face inversion (see Rossion and Gauthier, 2002 for a review) or misaligning face-halves (Jacques and Rossion, 2010). Further, previous reported N170 repetition suppression effects in race processing implicate an early stage of perceptual encoding whereby other-race faces are not encoded for identity as efficiently compared to same-race faces (Vizioli *et al.*, 2010a,b). The late N170 effects in our study may index a similar process of encoding including identity, aided through the perception of other-race visual signals in comparison to the less salient same-race signals.

At 30% coherence level, the late-N170 effect was no longer observable; instead, a very distinct occipito-temporal effect in the N250 time range clearly manifested in both hemispheres, which was also observed at the 40% coherence level, and at 50% and 60% although only in the right hemisphere. The transition from a late N170 effect to an N250 is quite interesting and invites speculation that the transition is rather categorical than gradual. Specifically, the N250 within the context of race is theorized to reflect the greater effort required when individuating other-race faces (Herzmann, 2016). The transition from an N170 effect to an N250 effect may reflect the greater amount of visual information available in the 30+% coherence levels, whereas the 20% coherence level may be sufficiently degraded to remove the ability to perceive characteristic individuating features while retaining the visual signals necessary for race categorization. The influence of individuation processes on the N250 has been observed in a series of ERP studies focusing on race, where other-race faces have been found to elicit greater negative activation than same-race faces (Sun *et al.*, 2014; Wiese *et al.*, 2014; Herzmann, 2016). Caldara *et al.* (2004) interpreted the later activation differences relating to race processing as reflecting the interplay between race perception and differences in the degree of semantic representations engendered by same- and other-race faces. Viewed within the framework of face and race perception advanced by Valentine and Endo (1992), the greater experience with same race faces produces a broader and more elaborated face-space representation area compared with other-race faces, which are clustered more tightly due to the increased salience and thus apparent similarity of the visual signals characteristic of different races. The enhanced N250 activation observed in our study in the higher coherence levels (as well as in preceding studies utilizing full-structure faces) may thus reflect the individuating differences inherent when encountering same- and other-race faces. However, the earlier N170 effect at 20% would appear more likely to reflect a lower-level perception that ignites individuation. In other words, the perception and categorization of race does not intrinsically require individuation processes, but rather relies on the perception of visual signals preceding this processing stage which may also modulate later processes. In terms of timing, this interpretation is in line with the behavioural results of de Lissa *et al.* (2021) which used peripheral presentations of same- and other-race faces, finding reliable race categorization to take place within 200 ms of presentation. Such remarkable speeds implicate very early stages of face processing. Indeed, at 60% and 100% coherence levels, a very early P1 effect was observed, with higher amplitudes for other- than same-race faces (Figures 9 and 10). While the P1 is an inconsistent index of race processing (see Serafini and Pesciarelli, 2023 for review), some studies have found an enhancement for other-race faces (Anzures and Mildort, 2021), and have been interpreted as reflecting differences in how people process configural information in same- and other-race faces (Hahn *et al.*, 2012; Wang *et al.*, 2020). The theoretical framework for such an effect was advanced by Zhao and Bentin (2011) as being due to enhanced use of configural information in individuation processes in same- relative to other-race faces, while not a process specifically necessary for race-categorization processes. It is of particular interest to the current study that these effects only manifested in response to visually clear and structurally intact faces, and were eroded as phase-coherence was further degraded.

In fact, the sequence of effects observed in the current study implicates a series of processes that are affected as visual quality is degraded. At full and partial phase coherence levels, the differences are very early P1, and not so substantial in voltage magnitude. As image quality is lost, a later N250 effect emerges, corresponding to when individuation processes would typically be expected to occur. And finally, a behavioural and ERP threshold is observed where enhanced sensitivity to other-race faces is at a threshold and late N170 effects emerge. These results suggest that there may be varying visual cues for race at varying levels of visual quality. However, it is important to note that the nature of these visual cues is not clarified in the current study. The specific differences in physiognomy that people rely on to categorize race might interact with visual quality to produce an asymmetry in how different race faces are impacted by visual degradation. Such a consideration may move closer to understanding how the specifics of visual cues used in race categorization may change according to the different race faces presented. In line with this concept, further investigation should be directed at how other visual cues might be compromised through visual stimulus degradation, such as dominance, aggressiveness, etc. We did not control for these dimensions in our stimuli set; however, the possibility for these to interact with perception of race at varying levels of visual degradation is an important thing to consider, given our results. For example, visually degrading faces through Gaussian filtering has been observed to lead to enhanced ratings of attractiveness (Sadr and Krowicki, 2019), which has also been linked to race and health perception using morphed ‘mixed race’ composites (Rhodes *et al.*, 2005). Given that the current behavioural and neural results point to enhancements in sensitivities to other race faces as a function of how much visual information is contained in the face images, the race-in-noise paradigm seems well-suited in investigating the relationships these other factors might have with race perception in visually degraded stimuli, as well as factors that are independent.

## Conclusions

These results may have strong implications for how stimulus quality—the available information—may impact various stages of processing, from early face categorization through to individuation. Race can be extracted at different latencies in the time course and ERP components at the expense of the granularity of categorization, from a very early gross estimation (i.e. P1) to more elaborate (i.e. N170) and rich representations (i.e. N250). As such, the categorical perception of race cannot be considered as one unique process, but rather may be performed at various perceptual levels. In addition to this observation, the speed at which race is perceived and acted upon in the brain, or more specifically the very early enhanced sensitivity to other-race faces, may shed light on the way in which faces are processed and represented cognitively. Valentine and Endo’s (1992) multidimensional space framework accounts for differences in race categorization due to the relatively less-elaborated space for other-race faces, where the novel visual signals characteristic of particular races become a salient common link between them. The low-level nature of these visual signals is attested in behavioural findings showing that the other-race categorization advantage relies heavily on low-spatial frequencies, where the biggest accuracy and RT advantage is apparent to faces that were filtered to retain only low spatial frequencies (Zhang *et al.*, 2017). Given that these effects are cross-cultural and not likely due to any inherent visual differences between faces of different races, these low-level visual cues differ only in the weight of their representations in our brains. Future studies may then focus specifically on how such weights might change within a testing session and across changes in experience with faces of other-races, as well as to incorporate the likelihood of these factors interacting with varying types of implicit biases and prejudice (Rösler and Amodio, 2022). Additionally, future functional imaging studies may benefit by manipulating face signal-intensity to isolate neural structures related to varying types of visual race-analysis. It has recently been shown that race-categorization (and the ORCA itself) may be preserved in acquired prosopagnosia where identity recognition is otherwise significantly impaired (Schaller *et al.*, 2023). Further elucidation of the overlap and separation of low-level visual analysis with higher-order individuation processes in race categorization would provide useful insight into how such a dimension is stored and accessed in the brain. The interplay between early perception and categorization of race and later individuating processes may thus be more fully explored through utilizing the race-in-noise paradigm, and the apparent behavioural and neural threshold effects observed in the current study may serve as useful points of comparison.

## Data Availability

All data used in statistical analyses and represented in figures are available online on the Open Science Framework repository at https://osf.io/sx6b2.
